# Caregiver dynamics and factors affecting health-seeking behaviors for childhood diarrhea in Mukuru informal settlements in Nairobi, Kenya

**DOI:** 10.3389/fpubh.2025.1670985

**Published:** 2025-11-21

**Authors:** Rahma Osman, Eric Ndombi, Mary Gitahi, Amos Njuguna, Mackwellings Phiri, Amha Mekasha, Nigel Cunliffe, Chisomo Msefula, Khuzwayo C. Jere, Daniel Asrat, Scholastica Kamwethya, Morgan Wasilwa, Jessicah Jepchirchir, Deborah Nyirenda, Beatrice Ongadi, Phelgona Otieno, Chikondi Mwendera, Kelvin Kering, Cecilia Mbae, Samuel Kariuki, GHRG-GI Consortium, Prisca Benedicto, Christina Bronowski, Jobiba Chinkhumba, Helen Clough, Jen Cornick, Neil French, Dan Hungerford, James Ngumo Karis, Siobhan Mor, Steven Sabola, Edson Mwinjiwa, Latif Ndeketa, Virginia Pitzer, Yemisrach Shumeye, Abebe Habtamu Tamire, Fred Were, Mengistu Yilma, Christine Kioko, Winfred Mbithi, Cheryl Giddings, Michael Muraya, Eunice Njoki, Salome Ngamau

**Affiliations:** 7 Malawi Liverpool Wellcome Programme, Blantyre, Malawi; 8 University of Liverpool, Liverpool, United Kingdom; 9 Kamuzu University of Health Sciences, Blantyre, Malawi; 10 Malawi Liverpool Wellcome Programme, Blantyre, Malawi; 11 Kenya Medical Research Institute, Nairobi, Kenya; 12 Shewit Weldegebriel, Addis Ababa University, Addis Ababa, Ethiopia; 13 Yale, New Haven, United States; 14 Addis Ababa University, Addis Ababa, Ethiopia; 1 Department of Family Medicine, Community Health and Epidemiology, Kenyatta University, Nairobi, Kenya; 2 Centre for Microbiology Research, Kenya Medical Research Institute, Nairobi, Kenya; 3 Malawi Liverpool Wellcome Programme, Blantyre, Malawi; 4 Addis Ababa University, College of Health Sciences, Department of Paediatrics and Child Health, Addis Ababa, Ethiopia; 5 Liverpool School of Tropical Medicine, Pembroke Place, United Kingdom; 6 Institute of Infection, Veterinary and Ecological Sciences, University of Liverpool, Liverpool, United Kingdom

**Keywords:** healthcare seeking behavior, caregivers, diarrhea, children under 5 years, urban informal settlement, Kenya

## Abstract

**Background:**

Diarrheal disease is the third leading cause of morbidity and mortality in children under 5 years globally. Approximately 1.7 billion diarrheal cases are reported annually resulting in more than 400,000 deaths in children under five. Sub-Saharan Africa still harbors populations with the highest global mortality rates for diarrhea accounting for approximately 60% of all diarrheal deaths in this age group. In Kenya, diarrhea significantly contributes to child mortality, with around 16,000 deaths annually among children under five, particularly affecting those living in urban informal settlements where access to clean water and sanitation is often limited. This study sought to investigate the determinants of healthcare-seeking practices among caregivers of children under 5 years with diarrhea in Mukuru informal settlement in Nairobi County.

**Methods:**

This study employed a cross-sectional design to investigate healthcare-seeking behaviors among caregivers of children under 5 years with diarrhea. Between September 2023 and January 2025, we conducted focused group discussions among 90 caregivers of children identified through purposive sampling. In addition, using a structured questionnaire, we interviewed 374 caregivers at the Mother and Child Health (MCH) clinics at three health facilities, targeting those who brought healthy children for immunization. Qualitative data were analyzed thematically using NVivo while bivariate and multivariate analysis was performed on the quantitative data.

**Results:**

Caregivers with secondary education had lower odds of seeking care compared to those with only primary education (AOR = 0.171, 95% CI: [0.060–0.488], *p* = 0.001). Additionally, caregivers who perceived that poor human fecal disposal causes diarrhea had significantly higher odds of seeking healthcare (AOR = 3.259, 95% CI: [1.185–8.965], *p* = 0.022). Trust in clinicians also played a role, with those who strongly agreed about the importance of clinicians in treating diarrhea having higher odds of seeking care (AOR = 0.259, 95% CI: [0.028–2.374], *p* = 0.050).

**Conclusion:**

This study demonstrates that specific socio-demographic factors, particularly education level and perceptions of disease causation, significantly influence health-seeking behaviors among caregivers of children under five with diarrhea in Mukuru informal settlement. Caregivers with secondary education were less likely to seek care, while those aware of the link between poor sanitation and diarrhea showed increased healthcare-seeking behavior. Additionally, trust in healthcare providers was identified as a crucial factor affecting care-seeking decisions.

## 1 Introduction

Diarrheal diseases are a leading cause of morbidity and mortality in children under 5 years globally (1). According to the World Health Organization (WHO) an estimated 1.7 billion diarrheal cases are reported annually among children under 5 years, resulting in more than 400,000 deaths globally (2). Sub-Saharan Africa bears the highest under 5 mortality rates due to diarrhea accounting for approximately 60% of all diarrheal deaths in this age group (3).

Several African countries, such as Ethiopia, Kenya, Niger, and Côte d’Ivoire, face a high prevalence of diarrhea, which is linked to significant morbidity and mortality in sub-Saharan Africa, (4–6). In Kenya, diarrhea is a significant public health challenge affecting millions of children under 5 years and is among the leading causes of death in this population. In 2018, Kenya had 1,499,146 cases of diarrhea in children below 5 years (5). Of these cases, 136,028 were from Nairobi. The mortality rate for diarrheal diseases in children under five is approximately 4.5% in Kenya, equating to about 67,000 deaths annually, while in Nairobi’s informal settlements, it can reach up to 8%. A study in Mathare established that there is a high prevalence of diarrheal disease in informal settlements of Nairobi, in particular among children under 5 years, with a previous study reporting a prevalence of 18.7% (5). Dense population, poor water, sanitation and hygiene infrastructure characterize informal settlements, which are likely to contribute to the high burden of diarrheal diseases (7). Diarrhea is, therefore, a prominent public health problem in Nairobi’s informal settlements and a major contributor to child mortality (5). Effective management of diarrhea is essential for effective control of its complications, including dehydration and death, but few cases are treated. The World Health Organization (WHO) recommends oral Rehydration Salts (ORS), zinc supplements, and continued feeding as the primary care approach for acute diarrhea (8). Healthcare seeking behavior is an action taken by an individual in response to an external stimulus to find a suitable solution after a child has fallen ill (9). While appropriate healthcare seeking could reduce disease severity and mortality associated with diarrhea, caregivers sometimes delay or forgo seeking effective medical care (10). Several factors including socio-economic, cultural, and health care systems have been reported to influence healthcare seeking behaviors among caregivers of children (11–13). Despite the identification of various factors influencing healthcare-seeking behaviors, there remains a significant gap in understanding how these factors manifest within the unique context of informal settlements. Most existing studies overlook the distinct socio-economic and cultural dynamics that affect caregivers in urban informal settlement like Mukuru, failing to capture the specific barriers and local beliefs that influence health-seeking practices. This study is informed by the Health Belief Model, which emphasizes the role of individual perceptions and external factors in health-related behaviors We aimed to understand the determinants of healthcare seeking behaviors among caregivers in Mukuru Informal settlement. This information would be critical in the formulation of economically viable, socially and culturally acceptable strategies of reducing the burden of diarrheal diseases.

## 2 Methods

### 2.1 Study design

This study employed a cross-sectional design, integrating both qualitative and quantitative approaches. The mixed-methods framework allowed an in-depth exploration of health system and socio-demographic factors, caregiver perceptions and barriers influencing healthcare-seeking practices for diarrheal illnesses in children under 5 years.

### 2.2 Study setting and population

The study was conducted in the Mukuru informal settlement in Nairobi. Mukuru informal settlement is located approximately 15 km from the city centre and is characterized by dense population, poor quality housing, high poverty levels, limited access to clean water, poor sanitation and hygiene practices. The study participants were caregivers of children below 5 years of age.

### 2.3 Data collection

Data were collected using both quantitative and qualitative methods between September 2023 and January 2025. For quantitative data, structured interviews were conducted with 374 caregivers at the Mother and Child Health (MCH) clinic, specifically targeting those who brought healthy children for immunization. The inclusion criteria for the study required participants to be residents of Mukuru, have children under 5 years old, be able to communicate effectively, and demonstrate a willingness to participate. Conversely, the exclusion criteria included caregivers who visited the hospital but did not reside in Mukuru, as well as those who brought sick children to the hospital. The latter group was excluded to avoid interrupting their ongoing healthcare-seeking process. The study was conducted at three health facilities; Mukuru Health Centre, Maendeleo Health Centre, and MMM Hospital where data was collected at different times. The most recent version of CommCare, an open-source mobile data collection platform, was utilized for this purpose. For qualitative data, nine focus group discussions (FGDs) were organized, corresponding to the nine distinct zones within the Mukuru informal settlement. This approach ensured that diverse perspectives from various areas were captured. Members of the Community Engagement and Involvement (CEI) teams, who were selected from different zones, played a crucial role in mobilizing residents. They approached caregivers who had children under 5 years, ensuring that participants were available and willing to share their experiences. Each FGD consisted of 6–8 participants, fostering an environment conducive to open discussion. Guiding questions were translated from English to Swahili, and responses were recorded using a voice recorder to ensure accurate transcription, with supporting notes taken to complement the audio recordings.

### 2.4 Data analysis

Descriptive and inferential statistics were used to analyze the quantitative data. Descriptive statistics, such as frequency counts and percentages, were used to describe the characteristics of the sample of the respondents. Bivariable analysis was conducted to assess the association between the dependent variable, health-seeking behavior, and independent variables, which were tested using binary logistic regression. To evaluate multicollinearity among the independent variables, the variance inflation factor (VIF) was calculated, resulting in the exclusion of predictors with VIF values exceeding 5 from the initial model. Subsequently, a multivariate logistic regression analysis was performed, incorporating several predictor variables from the binary logistic model. These variables were assessed simultaneously in a single block to evaluate their predictive ability while controlling for the effects of other predictors in the model (14). Adjusted odds ratio (aOR) and 95% confidence interval are reported to establish determinants of health seeking practice. Audio recordings of the focus groups were transcribed verbatim, translated into English, and thematically analyzed using NVIVO 14 software (15). A codebook was generated through both inductive and deductive coding.

### 2.5 Variables

In this study, the dependent variable was defined as “health care seeking practice.” This variable was classified into two distinct categories: appropriate and inappropriate health care seeking. Appropriate practices included taking a child with diarrhea to a health facility or administering oral rehydration solution (ORS) or zinc supplements. On the other hand, inappropriate health care seeking covered actions such as purchasing medications from a pharmacy, consulting a traditional herbalist, using leftover medications from home, or opting not to seek any medical intervention. The independent variables used in the study are socio-demographic factors, health system factors, knowledge and perceptions of caregivers and barriers to healthcare seeking.

### 2.6 Ethical consideration

The study obtained approval from KEMRI’s Scientific and Ethics Review Unit (SERU) (No: KEMRI/SERU/CMR/P00267-012-2023/4946). Written, informed consent was secured from caregivers of children under five before conducting focus group discussions and interviews, ensuring participants understood the study’s purpose and their rights. Participation was voluntary, with assurances that names would not be recorded to maintain confidentiality.; Participants were referred to as respondents in transcriptions. Audio recordings were solely used for research purposes and were not shared with unauthorized individuals. This work forms part of the NIHR Global Health Research Group on Gastrointestinal Infections (GHRG-GI) at the University of Liverpool (Central University Research Ethics Committee D, #12443).

## 3 Results

### 3.1 Common childhood illnesses among children under five in Mukuru

The study revealed that diarrhea is very common among children under five in the Mukuru informal settlement, with 77.8% of caregivers reporting recent episodes of diarrhea in their children. Similarly, the theme on diarrhea disease burden emerged strongly echoing this quantitative finding as illustrated by the following quote from one focus group discussion:

“*The disease that mostly affects our kids is diarrhea. We live near drainages; there are plots and open sewers everywhere. All the plots drain dirty water into open sewers. Most of our kids’ playing items always fall into the open sewers, and they still pick them up and play with them.”* (R1, FGD_002).

*I consider cholera as the most burdening disease in Mukuru informal settlements. The water we use for drinking is not clean and my child suffered from cholera just the other day. I had to rush her child to the hospital after several episodes of diarrhea. R1, FGD_001.*

*Cholera and diarrhea are the diseases that are most burdensome in this community. This is because when a child has diarrhea, they become weak and dehydrated very quickly. As a parent, if you do not have any knowledge on helping the child then there is a high chance of you losing them. R3, FGD_009.*

*Diarrhea and coughing are significant concerns in our community due to the risk of contamination from burst pipes. The bacteria responsible for diarrhea may contaminate the water pipes, posing a threat to the health of everyone in the community. This is particularly concerning when it comes to washing groceries and consuming untreated water. R4, FGD_006*.

### 3.2 Sociodemographic characteristics

Virtually all of the 374 participants interviewed (99%) were women, and the majority (81%) of the caregivers interviewed were married, while a significant proportion (74%) was unemployed Table 1. The level of education varied, with 59% having secondary education, 21% having primary education, and 20% possessing tertiary education. The distribution of children by gender was fairly balanced, with 56% being male. Less than half (41%) of the children were firstborns.

**Table 1:** Socio-demographic characteristics.

Variable	Categories	Frequency *n* (%)
Child’s gender	Male	209 (55.88)
	Female	165 (44.12)
Birth order of the child	First	152 (40.64)
	Second	130 (34.76)
	Third and above	92 (24.60)
Marital status of caregiver	Single	72 (19.25)
	Married	302 (80.75)
Educational status of the caregiver	Primary	78 (20.86)
	Secondary	220 (58.82)
	Tertiary	76 (20.32)
Caregivers’ occupation	Unemployed	275 (73.53)
	Employed	16 (4.28)
	Self-employed	83 (22.19)

### 3.3 Caregivers’ first responses to diarrhea

**Figure 1:**
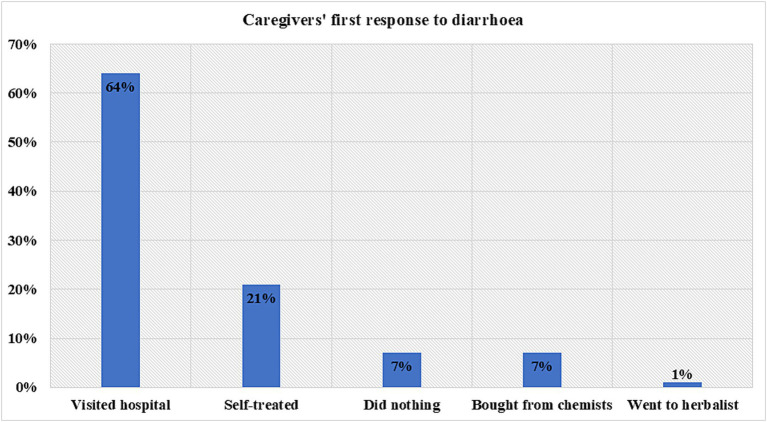
Caregiver’s first response to child’s diarrhea.

When caregivers were asked about their first responses to diarrhea in children, 64% said they sought care at a health facility as their initial action (Figure 1). Additionally, 21% opted for self-treatment at home, while 7% purchased medication without a prescription. Around 7% of the participants mentioned that they did nothing, anticipating that diarrhea would resolve on its own, while 1.0% of caregivers sought traditional remedies. These findings are supported by the following excerpts from the focus group discussions:

“When my child starts to have diarrhea, I give her boiled rice water when it doesn’t stop, I prepare wheat flour porridge. It helps so much. If it persists in now take her to hospital.” R3, FGD_002.

“According to other mothers who help us in the community, you take some sugar and salt and mix it with warm water then give it to the child. Then you observe the child….” R2, FGD_008.

“There is a tree that has roots back in my rural area. The roots of the tree are taken and crushed. It is then mixed with water and another herbal medicine is chewed by someone and then applied on the child. This then stops the diarrhea.” R1, FGD_009.

“Sometimes I might borrow from my neighbor any medicine they may have especially if I get sick at night. If I get better in the morning then I do not bother about it but if I don’t I go to the hospital.” (R8, FGD_009).

"I always have antibiotics at home; I never lack Amoxil or paracetamol because of my small children." (R2, FGD_010).

### 3.4 Association between socio-demographic characteristics and healthcare-seeking practices

Analysis of the association between socio-demographic characteristics and healthcare-seeking practices revealed married caregivers had a higher odds of seeking appropriate healthcare, although this was not statistically significant (cOR 1.666, *p* = 0.085). The occupation of the caregiver significantly influenced health-seeking behaviors, with self-employed caregivers showing a higher odds ratio of 1.84 and a *p* = 0.038 (Table 2). The results are corroborated by findings from qualitative data in the following excerpts:

**Table 2:** Association between socio-demographic characteristics and healthcare-seeking practices.

Variable	Category	Appropriate health seeking behavior (%)	Inappropriate health seeking behavior (%)	Crude odds ratio	*p*-value
Child’s gender	Male (Ref)	105 (56.45)	59 (56.19)	1	–
	Female	81 (43.55)	46 (43.81)	0.989	0.966
Birth order	First (Ref)	63 (33.87)	43 (40.95)	1	–
	Second	68 (36.56)	39 (37.14)	1.19	0.537
	Third and above	55 (29.57)	23 (21.90)	1.632	0.123
Marital status	Single (ref)	32 (17.20)	27 (25.71)	1	–
	Married	154 (82.80)	78 (74.29)	1.666	0.085
Educational status	Primary (ref)	45 (24.19)	17 (16.19)	1	–
	Secondary	101 (54.30)	68 (64.76)	0.561	0.075
	Tertiary	40 (21.51)	20 (19.05)	0.756	0.478
Caregiver’s occupation	Unemployed (ref)	118 (63.44)	80 (76.19)	1	–
	Employed	11 (5.91)	4 (3.81)	1.864	0.3
	Self-employed	57 (30.65)	21 (20.00)	1.84	**0.038**

Bold value indicates statistically significant.

*“When you come with your husband you are attended to faster than everyone else, especially during immunization” FGD 002.*

*“The cost of medications, consultation fees, and other healthcare expenses hinder those of us who are unemployed from receiving the services.” R6, FGD_005.*

*……….if I do not have enough money to cater for the dosage prescribed, then I am given a dosage that fits within my budget. If I get better before completing the doctor’s prescribed dosage, I do not bother to go back for the remaining dose, but if I do not get better, I continue buying within my budget. R1, FGD_009.*

### 3.5 Association between health system factors and healthcare-seeking practices

The proximity to health facilities was a factor influencing caregivers’ health-seeking behaviors. Caregivers living within 15 min of a health facility tended toward greater health-seeking practices, but this association was not statistically significant (cOR 0.086, *p*-value = 0.583). Additionally, caregivers who preferred private hospitals indicated better health-seeking practices compared to those who preferred government facilities, although this difference also lacked statistical significance (cOR 1.804, p-value = 0.073). Morever, those who had difficulty in paying for diarrhea treatment had 0.64 less chance of seeking medical healthcare compared to those who had easy in paying for the treatment (cOR = 0.641, *p* = 0.161) Overall, none of the associations presented in Table 3 reached statistical significance. These results are supported by findings from group discussion as shown by the following quotes:

**Table 3:** Association between health system factors and healthcare-seeking practices.

Variable	Category	Good (%)	Poor (%)	Crude odds ratio	*p*-value
Health facility near home	Government (ref)	132 (70.97)	66 (62.86)	1	–
	Private	54 (29.03)	39 (37.14)	0.692	0.155
Preferred health facility	Government (ref)	143 (76.88)	90 (85.71)	1	–
	Private	43 (23.12)	15 (14.29)	1.804	0.073
Distance to health facility	<15 min (Ref)	93 (50.00)	47 (44.76)	1	–
	15–30 min	79 (42.47)	46 (43.81)	0.868	0.583
	>30 min	14 (7.53)	12 (11.43)	0.59	0.222
Cost of treating diarrhea	Easy to pay (ref)	159 (85.48)	83 (79.05)	1	–
	Difficult to pay	27 (14.52)	22 (20.95)	0.641	0.161

*“Proximity to community health centers/clinics is a significant enabler. Since they are easily accessible by community members, it leads to seeking professional medical advice and treatment for diarrhea.” (R5, FGD_005).*

*“I prefer the R hospital (Private health centre) In fact, even if it’s a clinic, even if it’s taking weight measurements. They have good services and they are more concerned than public hospitals. So, I love R hospital even if I have to pay, I love it.” (R4, FGD_001).*

*“I live in Ruben. The hospitals around me are X and Y. I go to Y which is a public hospital. I am not charged anything there everything is free apart from the unavailable medicines at the facilities which I am sent to buy from outside. The lab tests are also free. I access these facilities by foot.” (R8, FGD_008).*

*“My child was sick, I took her to the hospital and I was just given the prescription so that I can purchase it from a private chemist. One was going for 800 Kenyan shillings (6.19 USD) and I am supposed to buy 3 totaling to 2,400 Kenyan shillings (18.58 USD). I did not have such an amount of money. I just went to the nearby chemist and bought Amoxil of a day’s dose.” (R10, FGD_001).*

### 3.6 Association between knowledge, attitudes, and perceptions and healthcare-seeking practices

In terms of knowledge, attitudes, and perceptions regarding diarrhea, the majority of caregivers (92.25%) reported awareness of the signs and symptoms of diarrhea (cOR = 0.38, *p*-value = 0.113). Additionally, 83.50% of the participants knew about Oral Rehydration Salt (ORS) (cOR = 0.408, *p* value = 0.005). However, a prevalent misconception emerged, with 85.83% believing that teething causes diarrhea, although statistically this was not significant (cOR = 1.114, *p* value = 0.863) (Tables 4, 5).

**Table 4:** Association between knowledge, attitudes, perceptions, and healthcare-seeking practices.

Variable	Category	Good (%)	Poor (%)	Crude odds ratio	*p*-value
Diarrhea affects bottle-fed children	Agree (Ref)	107 (57.53)	58 (55.24)	1	–
	Neutral	28 (15.05)	26 (24.76)	0.584	0.09
	Disagree	51 (27.42)	21 (20.00)	1.316	0.369
Teething causes diarrhea	Agree (Ref)	167 (89.78)	93 (88.57)	1	–
	Neutral	8 (4.30)	4 (3.81)	1.114	0.863
	Disagree	11 (5.91)	8 (7.62)	0.766	0.58
Liquid foods aggravate diarrhea	Agree (Ref)	76 (69.09)	34 (32.38)	1	–
	Neutral	29 (26.36)	28 (26.67)	0.463	**0.022**
	Disagree	5 (4.55)	43 (41.90)	0.843	0.541
Poor human feces disposal	Strongly Agree (Ref)	151 (81.65)	85 (80.95)	1	–
	Agree	33 (17.84)	16 (15.24)	1.161	0.654
	Disagree	2 (1.08)	4 (3.81)	0.281	**0.148**
Diarrhea healthcare services	Strongly Agree (Ref)	154 (82.80)	71 (67.62)	1	–
	Agree	30 (16.13)	19 (18.10)	0.728	0.331
	Neutral	2 (1.08)	15 (14.29)	0.061	**<0.001**
Clinicians treat diarrhea	Strongly Agree (Ref)	157 (84.41)	68 (64.76)	1	–
	Agree	27 (14.52)	28 (26.67)	0.418	**0.004**
	Disagree	2 (1.08)	9 (8.57)	0.096	**0.003**
Taking child for diarrhea treatment	Strongly Agree (Ref)	154 (82.80)	69 (65.71)	1	–
	Agree	32 (17.20)	31 (29.52)	0.463	**0.008**
	Disagree	0(0)	5(4.76)	–	–

Bold value indicates statistically significant.

**Table 5:** Association between knowledge, attitudes, perceptions, and healthcare-seeking practices.

Variable	Category	Good (%)	Poor (%)	Crude odds ratio	*p*-value
Handwashing prevents diarrhea	Strongly agree (ref)	159 (85.48)	84 (80.00)	1	–
	Agree	27 (14.52)	20 (19.05)	0.713	0.297
	Disagree	0 (0.00)	1 (0.95)	(empty)	–
Do you know signs and symptoms?	Yes (ref)	181 (97.31)	98 (93.33)	1	–
	No	5 (2.69)	7 (6.67)	0.387	0.113
Do you know ORS?	Yes (ref)	164 (88.17)	79 (75.24)	1	–
	No	22 (11.83)	26 (24.76)	0.408	**0.005**
Where did you learn about ORS?	Medical prescription (ref)	147 (89.63)	59 (74.68)	1	–
	Consultation of pharmacists	5 (3.05)	6 (7.59)	0.334	0.08
	Family/Friends	12 (7.32)	14 (17.72)	0.344	**0.012**

Bold value indicates statistically significant.

*"I educate my family on ways of preventing diarrhea like boiling drinking water and proper handwashing." (R2, FGD_009).*

*“I usually give my child ORS and zinc when I see he/she has diarrhea, then watch over him/her. If it persists, I take him/her to a health facility” R2, FGD_005.*

*“I did not give her anything, this is because the diarrhea was caused by teething and I was told by neighbor that it will resolve on its own” R10, FGD_007.*

*“…………… Then there is a way during the teething process they may suffer from diarrhea and febrile episodes that do not end.” — R2, FGD_008.*

*“I have also heard of the evil eye theory and know that sometimes it is associated with X, Y or Z tribes. It is said that the evil eye from X tribe is the worst. The child has severe diarrhea and fever and if you take them to the hospital to get an injection they die. They should not be injected or taken to the hospital” (R8 FGD_009).*

### 3.7 Multivariate logistic regression model (AOR) (*p* < 0.2)

Multivariable logistic regression analysis identified key variables linked to healthcare-seeking behaviors as shown in Table 6. Caregivers with secondary education had lower odds of seeking care compared to those with primary education (AOR = 0.171, 95% CI: [0.060–0.488], *p* = 0.001). Additionally, caregivers who perceived that poor human fecal disposal causes diarrhea had significantly higher odds of seeking healthcare (AOR = 3.259, 95% CI: [1.185–8.965], *p* = 0.022). Trust in clinicians also played a role, with those who strongly agreed about the importance of clinicians in treating diarrhea having higher odds of seeking care (AOR = 0.259, 95% CI: [0.028–2.374], *p* = 0.050).

**Table 6:** Multivariate logistic regression model (AOR) (*p* < 0.2).

Variable	Categories	Odds ratio	*p*-value	95% CI
Marital status	Single (ref)	1	–	–
	Married	1.871	0.137	0.819–4.275
Educational status	Primary (ref)	1	–	–
	Secondary	0.171	**0.001**	0.060–0.488
	Tertiary	0.226	**0.019**	0.065–0.784
Caregiver’s occupation	Unemployed (ref)	1	–	–
	Employed	0.973	0.97	0.235–4.020
	Self-employed	1.994	0.083	0.914–4.348
Cost of treating diarrhea	Easy to pay (ref)	1	–	–
	Difficult to pay	0.898	0.805	0.384–2.102
Preferred health facility	Government (ref)	1	–	–
	Private	2.228	0.075	0.922–5.387
Health facility near home	Government (ref)	1	–	–
	Private	0.647	0.211	0.327–1.279
Diarrhea healthcare services	Strongly agree (ref)	1	–	–
	Agree	1.101	0.843	0.425–2.850
	Neutral	0.174	0.066	0.027–1.122
Clinicians treat diarrhea	Strongly agree (ref)	1	–	–
	Agree	0.387	0.05	0.149–1.001
	Disagree	0.259	0.232	0.028–2.374
Poor human fecal disposal	Strongly agree (ref)	1	–	Ref
	Agree	3.259	**0.022**	1.185–8.965
	Disagree	1	(Empty)	–
Do you know signs and symptoms?	Yes (ref)	1	–	–
	No	0.68	0.717	0.084–5.478

Bold value indicates statistically significant.

## 4 Discussion

Caregivers with secondary and tertiary education were less likely to seek appropriate healthcare. A probable reason for this trend is that more educated caregivers often utilize alternative sources of information that guide them in self-treatment, likely preferring these methods to avoid long wait times and medication stockouts. However, it is important to note that these alternative sources can sometimes lead to inappropriate healthcare seeking practices. This finding is similar to observations from two informal settlements in Nairobi which reported that caregivers who completed primary level or higher level of education were less likely to seek appropriate care for children under five with diarrhea, in the two settlements (16). This contrasts with reports from a study in Western Kenya which suggests that caretakers with formal education were more likely to provide oral rehydration solution and visit healthcare facilities for childhood diarrhea (10). Similarly, a study in Ethiopia concluded that maternal knowledge and education significantly influenced healthcare-seeking behavior for childhood illnesses (17).

Caregivers’ perceptions significantly influence their healthcare-seeking behaviors, particularly regarding the cause and management of diarrheal diseases. Caregivers who believe that poor human fecal disposal causes diarrhea exhibit significantly higher odds of seeking healthcare. Additionally, those who believe that clinicians play a crucial role in managing these conditions are more likely to seek professional care. This aligns with findings from previous studies, which emphasize the impact of perceived illness severity and trust in healthcare systems on caregivers’ decisions (18). Furthermore, caregivers who receive health education from healthcare staff often demonstrate improved healthcare-seeking practices (19). This can be attributed to the heightened confidence caregivers develop in the healthcare professional’s knowledge and their trust in the information provided during educational sessions. Such trust not only empowers caregivers to make informed health decisions but also fosters a collaborative relationship between caregivers and healthcare providers, ultimately enhancing the likelihood of seeking timely medical assistance for their children.

The study highlighted a prevalent belief among caregivers that teething causes diarrhea, a misconception is particularly concerning as it can influence caregivers’ health-seeking behaviors. The findings from the study indicate that most caregivers attributed diarrhea to teething, hence delayed care seeking. Although this association was not statistically significant, it emerged as a strong theme during focus group discussions, reflecting a deeply rooted perception among caregivers. This result is supported by Mengistie at al who reported that caregivers who perceived teething as the cause of diarrhea were less likely to provide ORT to their children than the caregivers who did not perceive teething as a cause of diarrhea. Similar misconceptions have been documented in Nigeria and Pakistan where the belief that teething contributes to diarrhea persists among caregivers (20, 21). Such misunderstandings can lead to delays in seeking appropriate treatment, as caregivers may underestimate the severity of diarrhea and rely on ineffective home remedies.

Barriers such as financial barriers emerged as a significant challenge to healthcare seeking behavior in this study especially during the focus group discussions, echoing findings from a study conducted in Tanzania which described financial costs as a challenge, especially in a population where the majority of people are uninsured (22). Financial barriers have been found to prevent poor populations from accessing healthcare in sub-Saharan Africa (23). Out-of-pocket payments for healthcare services are common, placing a heavy financial burden on families especially the ones living in informal settlements. However, a study on abolition of user fee conducted in Uganda suggests that while financial constraints are significant, cultural beliefs and norms can sometimes outweigh financial considerations, leading caregivers to prioritize traditional remedies over formal healthcare (24). Long waiting times in public health facilities also emerged as a significant concern, discouraging caregivers from seeking future care due to frustrations with congestion and inadequate service delivery, consistent with findings from study in coastal Kenya and Ethiopia (17, 25). Additionally, caregivers reported facing challenges in accessing essential medications at public hospitals, often resorting to local pharmacies because of persistent shortages. Similar observations were made from two studies in Kenya and Uganda which suggests that such shortages deter patients from seeking care and lead to reliance on potentially less effective home remedies (24, 25).

### 4.1 Study limitations

The study had some limitation which includes the possibility of recall bias, as caregivers reported instances of diarrhea and their subsequent actions based on their memories. Additionally, selection bias may be present due to the purposive and convenience sampling methods employed, which could affect the representativeness of the findings. Furthermore, social desirability bias may have influenced the responses of focus group discussion participants, who might have provided answers they believed were socially acceptable rather than expressing their true feelings. Another limitation is that the study was conducted in an informal settlement, which may limit the generalizability of the results to the broader population.

## 5 Conclusion

This study highlights that socio-demographic factors, particularly education level and perceptions of disease causation, significantly influence health-seeking behaviors among caregivers of children under five with diarrhea in Mukuru informal settlement. Caregivers with secondary education were less likely to seek care, while those aware of the link between poor sanitation and diarrhea were more proactive. Trust in healthcare providers also played a crucial role in care-seeking decisions. To enhance health outcomes, targeted educational interventions should improve caregivers’ understanding of causes of diarrhea and the importance of sanitation. Additionally, fostering trust through effective communication and quality care can encourage timely healthcare-seeking behaviors, ultimately benefiting children in Mukuru and similar informal settlements.

## Data Availability

The original contributions presented in the study are included in the article/supplementary material, further inquiries can be directed to the corresponding author.

## Group members of GHRG-GI consortium

The GHRG-GI comprises Prisca Benedicto, Malawi Liverpool Wellcome Programme, Blantyre, Malawi; Christina Bronowski, University of Liverpool, Liverpool, United Kingdom; Jobiba Chinkhumba, Kamuzu University of Health Sciences, Blantyre, Malawi; Helen Clough, University of Liverpool, Liverpool, United Kingdom; Jen Cornick, University of Liverpool, Liverpool, United Kingdom and Malawi Liverpool Wellcome Programme, Blantyre, Malawi; Neil French, University of Liverpool, Liverpool, United Kingdom and Malawi Liverpool Wellcome Programme, Blantyre, Malawi; Dan Hungerford, University of Liverpool, Liverpool, United Kingdom; James Ngumo Karis, Kenya Medical Research Institute, Nairobi, Kenya; Shewit Weldegebriel, Addis Ababa University, Addis Ababa, Ethiopia; Siobhan Mor, University of Liverpool, Liverpool, United Kingdom; Steven Sabola, Kamuzu University of Health Sciences, Blantyre, Malawi; Edson Mwinjiwa, Malawi Liverpool Wellcome Programme, Blantyre, Malawi; Latif Ndeketa, University of Liverpool, Liverpool, United Kingdom and Malawi Liverpool Wellcome Programme, Blantyre, Malawi; Virginia Pitzer, Yale, New Haven, United States; Yemisrach Shumeye, Addis Ababa University, Addis Ababa, Ethiopia; Abebe Habtamu Tamire, Addis Ababa University, Addis Ababa, Ethiopia; Fred Were, Kenya Medical Research Institute, Nairobi, Kenya; Mengistu Yilma, Addis Ababa University, Addis Ababa, Ethiopia; Christine Kioko, Kenya Medical Research Institute, Nairobi, Kenya; Winfred Mbithi, Kenya Medical Research Institute, Nairobi, Kenya; Cheryl Giddings; University of Liverpool, Liverpool, United Kingdom; Michael Muraya, Kenya Medical Research Institute, Nairobi, Kenya; Eunice Njoki, Kenya Medical Research Institute, Nairobi, Kenya; Salome Ngamau, Kenya Medical Research Institute, Nairobi, Kenya.
